# Living with or beyond lymphoma: A rapid review of the unmet needs of lymphoma survivors

**DOI:** 10.1002/pon.5973

**Published:** 2022-06-27

**Authors:** Vanessa Boland, Amanda Drury, Greg Sheaf, Anne‐Marie Brady

**Affiliations:** ^1^ School of Nursing & Midwifery Faculty of Health Sciences Trinity College Dublin Dublin Ireland; ^2^ School of Nursing Midwifery and Health Systems University College Dublin Dublin Ireland; ^3^ The Library of Trinity College Dublin Dublin Ireland

**Keywords:** blood cancer, cancer survivorship, haematological malignancy, Hodgkin, lymphoma, needs, non‐Hodgkin, psycho‐oncology, quality of life, unmet needs

## Abstract

**Objective:**

To establish an understanding of the unmet needs of people living with or beyond a lymphoma diagnosis. Survivors of lymphoma are at increased risk of unmet needs due to cancer, treatment‐related toxicities and extended survivorship. Despite the rapidly growing numbers of lymphoma survivors, their needs and research priorities are underserved and undervalued, therefore left largely unaddressed.

**Methods:**

A rapid review method and reflexive thematic analysis approach assimilated current knowledge. Eligibility criteria included quantitative, qualitative, or mixed approaches employing cross‐sectional, longitudinal, cohort or review designs focused on the needs of adult lymphoma survivors (any subtype or stage of disease). Five databases: CINAHL, EMBASE, Medline, PsycInfo and Scopus, were systematically searched.

**Results:**

Forty‐seven studies met the inclusion criteria via a stringent screening process facilitated by NVivo. Almost 60 per cent of articles were published within the last five years and investigated a homogenous lymphoma sample. Most studies employed quantitative approaches (77%) and cross‐sectional designs (67%). Studies were of high methodological quality. Five major themes were identified: disparity in health service delivery, the psychological impact of cancer, impactful and debilitating concerns, the monetary cost of survival and insufficient provision of survivorship information. A meta‐analytical approach was not feasible due to the breadth of methodologies of included studies.

**Conclusions:**

This review shows that lymphoma survivors experience a myriad of unmet needs across multiple domains, reinforcing the need for lymphoma‐specific research. However, more research is needed to advance and achieve informed decision‐making relating to survivorship care, placing due attention to the needs and research priorities of lymphoma survivors.

## 1 INTRODUCTION

### 1.1 Background

Haematological malignancies are a diverse group of cancers which develop in the blood‐forming tissue, including three broad categories: leukaemia, lymphoma, and myeloma.^1^ Haematological malignancies are distinct from solid tumours not only regarding their pathology but also in their potential presentation, treatment, progression and outcome.^1^, ^2^ These distinguishing factors contribute to an individualised experience from diagnosis to chronic effects, with the potential for varied needs to arise from these phases. Given the diversity amongst haematological malignancies, studies of heterogeneous samples of people living with and after haematological malignancies may be inappropriate. Subtype‐specific research is needed to ensure future care is responsive to survivors' needs.^3^, ^4^, ^5^

Therefore, this review is focused on lymphoma, the largest cohort of haematological malignancies originating in the lymphatic system and encompassing a variety of distinct disease entities.^2^ Given the widespread nature of the lymphatic system, lymphoma can affect any organ in the body with varying symptoms depending on where the cancer is.^6^ Lymphomas are categorised into two types – Hodgkin lymphoma (HL) and non‐Hodgkin lymphoma (NHL). Within the two subtypes, many variations exist. Compared with HL, NHL is more heterogenous with more than 60 subtypes and division into indolent or aggressive forms.^2^, ^7^ The varied clinical features and histological appearances of lymphoma present specific challenges such as difficult diagnosis, various management strategies and assorted prognoses.^7^, ^8^

The population of lymphoma survivors is substantially increasing thanks to the success of multimodal treatments.^9^ In 2020, there were an estimated 628,000 new lymphoma cases, with 283,000 new deaths worldwide for all age groups.^10^ Furthermore, the late effects of lymphoma and its treatments are critical determinants of long‐term morbidity, mortality and quality of life and can pose challenges to survivorship.^7^ Many lymphoma survivors will have extended life expectancies. Still, they may live with an increased risk for unmet needs resulting from late effects such as secondary malignancies, cardiac toxicity, pulmonary toxicity, and endocrine and gonadal dysfunction, which raise concerns about fertility.^2^, ^7^, ^11^

The concept of cancer survivorship is widely interpreted.^12^, ^13^, ^14^ However, it is broadly accepted that cancer survivorship begins at the time of diagnosis and continues until the end of life and can be referred to as ‘living with and beyond cancer’.^15^ Similarly, some debate and conceptual uncertainties exist on what constitutes a ‘need’ in healthcare.^16^, ^17^ The term ‘unmet needs’ distinguishes between concerns that survivors experience and wish for help in managing.^14^, ^17^, ^18^

Efforts to improve the availability of care and resources for cancer survivors have been advanced by assessing survivors' needs.^19^ The assessment of unmet needs enables the direct examination of an individual's perceived need for help. This allows for a more direct indication of required resources and the magnitude of the need for help, therefore facilitating the prioritisation of health services.^20^ At the same time, identifying higher‐level needs can quickly assist health care providers in recognising those most at risk and vulnerable.^21^ Quantifying the prevalence of survivors experiencing difficulties, including their unmet needs, is needed to promote recovery and supportive self‐management.^18^, ^22^, ^23^

Understanding the unmet needs of lymphoma survivors can enable informed decision‐making relating to survivorship care.^17^, ^22^, ^24^ Survivorship issues are of particular importance to this population with high survival rates.^6^ Yet, despite the rapidly increasing number of lymphoma survivors, their needs and research priorities rarely receive attention or are addressed.^25^, ^26^ Therefore, a rapid review has been undertaken to assess and assimilate current knowledge.^27^

### 1.2 Aims

This review aims to establish an understanding of the unmet needs of adults living with or beyond a lymphoma diagnosis using a rapid review method and reflexive thematic analysis approach. The research question asks, ‘*what are the unmet needs of people living with a diagnosis of lymphoma cancer*?’.

## 2 METHODS

### 2.1 Eligibility criteria

To be eligible for review, publications must refer to individuals with a lymphoma cancer diagnosis at any point of survival (from diagnosis onwards), any subtype or subgroup. Outcomes must refer to the needs of these individuals. Age criteria (more than half of the sample aged 18 years or older at the time of data collection, i.e., survey) applied focus to the needs of adult survivors as opposed to childhood or adolescence. Detailed inclusion and exclusion criteria are provided in Table 1.

**TABLE 1:** Inclusion and exclusion criteria in population exposure outcomes study design (PEOS) format

PEOS	Inclusion	Exclusion
Population	○ More than 50% of the sample were aged 18 years or older	More than 50% of the sample were aged 17 years or younger
	○ Lymphoma cancer diagnosis	
	○ Any subtype, subgroup, or stage of lymphoma cancer	
	○ At any point of survival (from diagnosis onwards)	
	○ Undergoing or completed any form of treatment resulting from a diagnosis of lymphoma cancer	
Exposure	○ Lymphoma cancer care/survivorship care	
Outcomes	Patient outcomes related to unmet needs which are a result of a diagnosis of lymphoma cancer include but are not limited to:	○ Studies testing the psychometric properties of patient health measures
	○ Survivorship care	
	○ Patient health outcomes	
	○ Late effects + consequences	
	○ Quality of life, patient wellbeing	
	○ Physical needs or concerns (symptom burden, fatigue)	
		○ Views of survivorship care
	○ Psychosocial needs or concerns (anxiety, depression)	
		○ Healthcare professionals or carers' experience or views of survivorship care
	○ Socioeconomic needs or concerns (financial burden)	
	○ Survivor information needs	○ Patient outcomes relating to childhood or adolescent survivors of lymphoma cancer
Study design	○ Systematic reviews	○ Individual case studies
		○ Intervention studies or RCTs
	○ Qualitative & Quantitative studies	○ Survival statistics
	○ Mixed‐methods studies	○ Pilot studies
	○ Population‐based studies	○ Opinion pieces ○ Editorials
	○ Prospective & retrospective studies	
		○ Commentaries
	○ Cross‐sectional studies	
	○ Longitudinal studies	
		○ Narrative literature review
	○ Grey literature (conference abstracts, reports, etc)	
Reporting	○ English language	○ Languages other than English
	○ Sufficient detail on population, outcomes, and results or appropriate detail on subgroup analysis	

### 2.2 Rapid review of the evidence

A rapid review method is a variation of a systematic review; this method was selected as it balances time constraints and available resources with considerations of bias.^27^ The literature on the needs of lymphoma survivors includes a broad range of evidence. Therefore, this review uses a streamlined approach to synthesise the available evidence.^27^, ^28^

### 2.3 Sources & searching

Five databases were systematically searched, including CINAHL, EMBASE, MEDLINE, PsycInfo and Scopus, in July 2021 and updated in February 2022. A restriction to literature published after 1 January 2006 was applied as the Institute of Medicine seminal report was published at this time^29^; this report has broadened the recognition of cancer survivorship in the cancer continuum resulting in progressive attention being placed on the unique challenges and needs of cancer and its subtypes.^30^ The search was filtered to the English language only. Authors were contacted to source full text when required. Reference lists and grey literature (i.e., conference abstracts) were examined for relevance to the eligibility criteria. However, abstracts lacked sufficient detail on population, outcomes, or results and were omitted. The search strategy and database searches were conducted by a researcher (Vanessa Boland) and an information specialist (Greg Sheaf). Key search terms related to ‘lymphoma’, ‘cancer survivors’, and ‘needs’ were searched; the resulting search string is outlined in Table A1.

### 2.4 Study selection

First, duplicate references were removed from the review library. Second, the title and abstract of each study were assessed for their relevance to the PEOS eligibility criteria. Two independent reviewers (Vanessa Boland and Amanda Drury) performed screening, supported by COVIDENCE software. To minimise any selection bias, discrepancies were resolved through discussion and required consensus from both reviewers. Finally, the full text of studies meeting the PEOS criteria was obtained for final screening by a single reviewer (Vanessa Boland). Secondary sources, anecdotal, opinion, editorials, clinical papers, case studies and case reports were excluded to minimise bias. As the goal of this review was to understand the experience of unmet needs for lymphoma survivors, rather than the effect of experimental interventions to improve outcomes, randomised controlled trials and intervention studies were also excluded.

### 2.5 Data abstraction

A reviewer conducted data abstraction (Vanessa Boland), and all studies received verification by a second reviewer (Amanda Drury & Anne‐Marie Brady). Data from studies were extracted using specific extraction templates as relevant to the quantitative, qualitative and review approaches included. Critical information was recorded, including authorship, country of origin, aim, design and method, recruitment method, instrument(s) used sample size and response rate. If reported, demographic information, such as sex, age, diagnosis, treatment, ethnicity/race, employment status, education level and partnership, were extracted.

### 2.6 Quality assessment

Methodological quality was assessed for all included studies using an appraisal tool developed by the Evidence for Policy and Practice Information and Coordinating (EPPI) Centre. Studies were evaluated according to the tool's twelve quality appraisal criteria (Table A2). One reviewer (Vanessa Boland) independently assessed each included study to establish to what extent each criterion was met, with independent verification of all judgements by a second reviewer (Amanda Drury & Anne‐Marie Brady) as guided by Cochrane's interim rapid review guidance.^28^ The twelve criteria measured the extent to which a study's findings provide a valuable contribution to the review.^31^

### 2.7 Data analysis

Reflexive Thematic Analysis (TA) formed the basis for this review's analysis due to its aptitude to amass and analyse the heterogeneous body of included literature by identifying patterns across the dataset.^32^ The first step of familiarisation involves a deep immersion and analytical engagement with the data. The literature is seen as data rather than as information. Next, a code labels something of interest within the data using analysis software (NVivo version 12.0). The dataset was assessed twice to provide robust and coherent coding. Steps three to five involved organising coded data into themes and developing rich data analysis, represented by themes.^32^

## 3 RESULTS

### 3.1 Summary of studies

**FIGURE 1:**
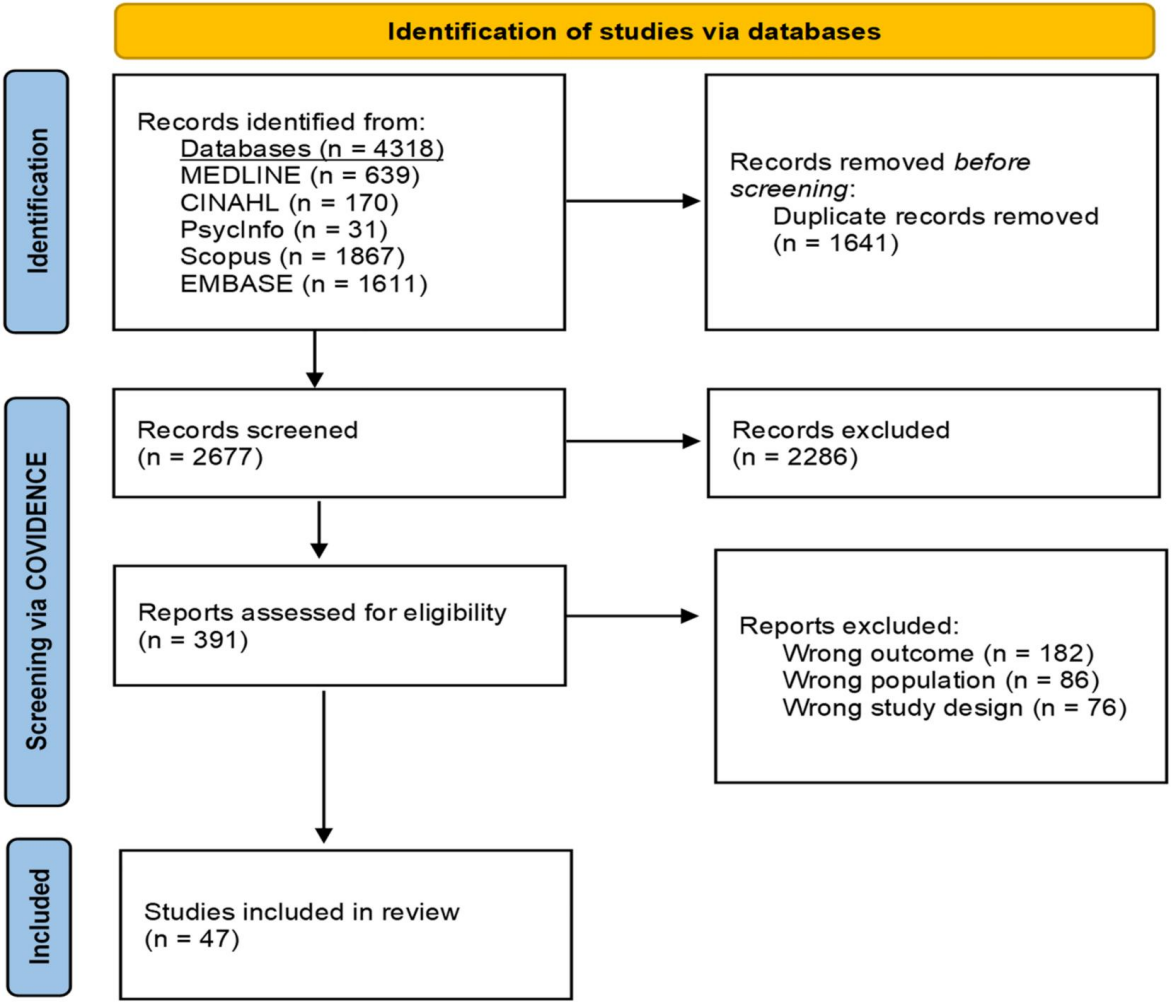
Preferred reporting items for systematic reviews and meta‐analyses (PRISMA)

Two thousand six hundred 77 studies were screened, with 391 requiring full‐text eligibility review (Figure 1). Forty‐seven studies met the inclusion criteria, of which 57% were published in the last five years. Most studies employed quantitative approaches (*n* = 36) and were more often cross‐sectional (*n* = 24). Studies originated from the United States (*n* = 15), Australia (*n* = 11), United Kingdom (*n* = 4), South Korea (*n* = 3) and multiple other countries located in Asia, Europe, and Oceania (Table 2). This reflects the current incidence rates of NHL, which are highest in Australia, New Zealand, Northern America and Europe.^10^ Over 30 per cent of included articles explicitly investigated unmet needs as the main outcome, while others explored closely related outcomes, such as quality of life (Table 2).

**TABLE 2:** Summary table of the characteristics of included articles (*n* = 47)

Characteristic	*n*	%
Methodology		
Quantitative	36	77
Qualitative	8	17
Review	3	6
Design		
Cross‐sectional	24	67
Longitudinal	8	22
Cohort	4	11
*Country		
United States	15	32
Australia	11	23
United Kingdom	4	9
South Korea	3	6
Germany	3	6
Other European countries	7	15
Other Asian countries	3	6
Other Oceania countries	1	2
Cancer site		
Lymphoma (total)	27	57
NHL and HL	12	26
NHL only	10	21
HL only	5	11
Mixed haematological malignancies (including >50% lymphoma)	18	38
Other cancers (including >50% lymphoma)	2	4
Method of data collection		
Questionnaire	36	77
Semi‐structured interviews	6	13
Focus groups	2	4
Systematic review approach	3	6
Outcomes assessed by included articles		
Unmet needs	15	32
Health‐related quality of life	8	17
Psychosocial	8	17
Quality of life	7	15
Post‐treatment experiences	4	9
Care experiences	3	6
Physiological	2	4

*Note rounding was used for figures, percentages for countries do not equate to 100 due to rounding.

Recruitment was mainly conducted via one or more cancer registries (*n* = 18) at hospitals, including single sites (*n* = 11), two sites (*n* = 4), three sites (*n* = 2) and only two studies employing online recruitment strategies (Table 2). For quantitative surveys, the most used instruments for assessing the needs (or associated outcomes, such as quality of life) of survivors included the European Organisation for Research and Treatment of Cancer Quality‐of‐life Questionnaire Core 30 (EORTC QLQ‐C30), Survivor Unmet Needs Survey (SUNS), Hospital Anxiety and Depression Scale (HADS) and the Functional Assessment of Cancer Therapy – General (FACT‐G) and Lymphoma subscale (FACT‐LYM).

Quantitative sample sizes ranged from small to exceptionally large. The smallest sample (*n* = 50) was specified for a young age group,^33^ and the largest sample (*n* = 4215) was a longitudinal study pooling data collected within clinical trials.^34^ The sample sizes of qualitative studies ranged from 6 to 51 (median = 17). A homogenous lymphoma‐specific population was investigated by most included studies (60%) (Table 2). Heterogenous samples mainly included other haematological malignancies, such as leukaemia and multiple myeloma.^3^, ^4^, ^21^, ^33^, ^35^, ^36^, ^37^, ^38^, ^39^, ^40^, ^41^, ^42^, ^43^, ^44^, ^45^, ^46^, ^47^, ^48^

Male participants were slightly more common. Most samples included participants of varying ages across adulthood (18–92 years), with four studies including participants aged 15 years or more.^21^, ^39^, ^49^, ^50^ Younger lymphoma survivors (<45 years) were explored by four others.^33^, ^51^, ^52^, ^53^ Older adults (65–85+ years) were only examined by one study.^35^ Chemotherapy was the most common treatment received by participants, with two studies focusing on outcomes relating to stem cell transplantation only.^36^, ^54^

Fourteen studies discussed the ethnicity or race of participants of these mainly White backgrounds were reported. Of the studies reporting participants' employment status, a sizeable percentage (40% – 85%) were employed. A medium level of education (secondary school or equivalent) of participants in over half of the studies reporting this sociodemographic factor (*n* = 24) was found, while participants mainly were partnered (range 59%–98%). However, the disparity in reporting acute or chronic survivorship, time since diagnosis, stage of disease, socioeconomic status and sociodemographic details of current literature inhibits further analysis to generate a more substantive understanding of this cohort.

### 3.2 Quality assessment

None of the included studies met all twelve quality criteria as this body of literature has not yet seen active involvement of participants in the design or conduct of studies as assessed by criterion L (Table A2). However, the literature included was of high methodological quality, with most studies (*n* = 38) meeting 11 of the 12 quality criteria. This was followed by 10 quality criteria being met by seven studies, and the lowest number of measures met was nine by two studies (Table A3).

Reporting of sociodemographic information of participants varied amongst studies. Several studies supplied rich sociodemographic data, such as sex, age, diagnosis, time since diagnosis, treatment received, ethnicity, education level, employment status, and marriage/partnership status.^21^, ^52^, ^55^, ^56^, ^57^, ^58^ These quantitative studies differed in recruitment strategies; some used multiple cancer or state registries,^21^, ^52^, ^56^ one used a national survivorship registry,^55^ others used three sites^57^, ^58^ and a single site.^59^ Reporting participants' age was missing from one study,^60^ while two other studies only provided the age, sex and diagnosis of participants,^26^, ^45^ limiting an adequate description of the study sample.

Studies using qualitative approaches made good attempts to show rigour and credibility of data collection methods or analysis. This included detailed reporting of methods (framework used, recording device, transcribed verbatim, data analysis software) and details regarding the refinement of theme development (i.e., analysis conducted by one or more researchers, use of comprehensive field notes, reflexive diary).^61^, ^62^, ^63^ One qualitative study required more detail in reporting data analysis as details about how the analysis was conducted, such as the number of researchers involved, the coding process or how subthemes to major themes were formed, were missing.^5^

Most quantitative studies adequately described statistical analysis (i.e., analysis of variance for comparing means of continuous variables). Most studies used validated and reliable tools (i.e., SUNS, FACT‐LYM), except Parry, Lomax,^44^ who used a tool that is not validated and Zucchetti, Bellini,^33^ who used a tool appropriate to assess the study's primary outcome of interest body image, yet not validated in the population of interest cancer survivors.

### 3.3 Reflexive thematic analysis

The active process of reflexive thematic analysis produced five significant themes relating to the needs of lymphoma survivors (Table 3). This includes disparity in health service delivery, psychological impact of cancer, impactful and debilitating concerns, the monetary cost of survival and insufficient provision of survivorship information. Study characteristics are outlined in Table A4.

**TABLE 3:** Theme development

Codes	Subthemes	Major themes
Health services Supports	Transition from patient to survivor Disruption to continuity of care Lymphoma care as a speciality service Health care provider support	Disparity in health service delivery
Psychological needs Emotional needs Social needs	Psychosocial needs Impaired cognitive functioning The impact of anxiety, depression & stress The fear of recurrence	Psychological impact of cancer
Physical needs Fatigue Symptoms	The continual burden of side effects during and after treatment Cancer‐related fatigue Barriers to social reintegration Physical implications of lymphoma	Impactful and debilitating concerns
Financial needs Work concerns	Financial implications The benefits of work Barriers to working	The monetary cost of survival
Information needs Practical needs	Inadequate information provisions Importance of communication from others Seeking survivorship advice	Insufficient provision of survivorship information

**TABLE A1:** Search string used in database searches

Terms relating to lymphoma
(MH ‘Lymphoma+’)
TI(Lymphoma*)
AB(Lymphoma*)
CI(Lymphoma*)
Terms relating to cancer survivorship
(MH ‘Cancer Survivors’)
TI(surviv* OR (after N2 cancer))/(W/2)/(NEAR/2)
AB(surviv* OR (after N2 cancer))
CI(surviv* OR (after N2 cancer))
Terms relating to needs
(MH ‘Needs Assessment’)/(DE ‘Needs Assessment’)
TI(((unmet OR unfulfil* OR overlook* OR perceived OR ‘supportive care’ OR physiological OR physical OR psychological OR emotional OR spiritual OR economical OR social OR psychosocial OR practical OR informational) N2 (need* OR concern*)) OR ‘late effect*’ OR ‘patient outcome*’)
AB(((unmet OR unfulfil* OR overlook* OR perceived OR ‘supportive care’ OR physiological OR physical OR psychological OR emotional OR spiritual OR economical OR social OR psychosocial OR practical OR informational) N2 (need* OR concern*)) OR ‘late effect*’ OR ‘patient outcome*’)
CI(((unmet OR unfulfil* OR overlook* OR perceived OR ‘supportive care’ OR physiological OR physical OR psychological OR emotional OR spiritual OR economical OR social OR psychosocial OR practical OR informational) N2 (need* OR concern*)) OR ‘late effect*’ OR ‘patient outcome*’)
Limitations
English language
Adults
Post Jan 2006 (IOM report)

**TABLE A2:** Quality appraisal criteria

Quality appraisal criteria
Quality of the study reporting
A = Aims and objectives clearly reported
B = Adequately described the context of the research
C = Adequately described the sample *and sampling methods*
D = Adequately described the data collection methods
E = Adequately described the data analysis methods
There was good or some attempt to establish the:
F = Reliability of the data collection methods*/tools*
G = Validity of the data collection methods*/tools*
H = Reliability of the data analysis methods
I = Validity of the results of the data analysis
Appropriateness of the methods
J = Used the appropriate data collection methods to allow for expression of views
K = Used the appropriate methods for ensuring the analysis was grounded in the views
L = Actively involved the participants in the design and conduct of the study

**TABLE A3:** Quality criteria met by included studies

Author	Design/Method	Quality criteria met	Total
Arboe et al (2017)	Cohort	A, B, C, D, E, G, H, I, J, K	10
Arden‐Close et al (2011)	Cohort	A, B, C, D, E, F, G, H, I, J, K	11
Arden‐Close et al (2010)	Systematic review	A, B, C, D, E, F, G, H, I, J, K	11
Beaven et al (2016)^84^	Cross‐sectional	A, B, C, D, E, F, G, H, I, J, K	11
Behringer et al (2016)	Longitudinal	A, B, C, D, E, F, G, H, I, J, K	11
Chen et al (2020)	Semi‐structured interviews	A, B, C, D, E, F, G, H, J, K	10
Chen et al (2012)	Cross‐sectional	A, B, C, D, E, G, H, I, J, K	10
Esser et al (2018)	Cohort	A, B, C, D, E, F, G, H, I, J, K	11
Fauer et al (2021)	Cross‐sectional	A, B, C, D, E, F, G, H, I, J, K	11
Georges et al (2020)	Cross‐sectional	A, B, C, D, E, F, G, H, I, J, K	11
Greaves et al (2014)	Cross‐sectional	A, B, C, D, E, F, G, H, I, J, K	11
Hackett & Dowling (2018)	Semi‐structured interviews	A, B, C, D, E, F, G, H, I, J, K	11
*Hall et al (2015)	Cross‐sectional	A, B, C, D, E, F, G, H, I, J, K	11
*Hall et al (2014)	Cross‐sectional	A, B, C, D, E, F, G, H, I, J, K	11
Hall et al (2013)	Cross‐sectional	A, B, C, D, E, F, G, H, I, J, K	11
Hernaes et al (2021)	Cross‐sectional	A, B, C, D, E, F, G, H, I, J, K	11
Herrmann et al (2020)	Semi‐structured interviews	A, B, C, D, E, F, G, H, I, J, K	11
Husson et al (2017)	Cross‐sectional	A, B, C, D, E, F, G, H, I, J, K	11
Kang et al (2018)	Longitudinal	A, B, C, D, E, F, G, H, I, J, K	11
Keegan et al (2012)	Cross‐sectional	A, B, C, D, E, F, G, H, I, J, K	11
Khimani et al (2013)	Longitudinal	A, B, D, F, G, H, I, J, K	9
Kim et al (2017)	Cross‐sectional	A, B, C, D, E, F, G, H, I, J, K	11
Kim et al (2014)	Cross‐sectional	A, B, C, D, E, F, G, H, I, J, K	11
Kreissl et al (2020)	Longitudinal	A, B, C, D, E, F, G, H, I, J, K	11
Lekdamrongkul et al (2021)	Cross‐sectional	A, B, C, D, E, F, G, H, I, J, K	11
Lobb et al (2009)	Cross‐sectional	A, B, C, D, E, F, G, H, I, J, K	11
Monterosso et al (2017)	Focus groups (two)	A, B, C, D, E, F, G, H, I, J, K	11
Murphy‐Banks et al (2022)	Semi‐structured interviews	A, B, D, E, F, G, H, I, J, K	10
Ng et al (2016)	Cross‐sectional	A, B, C, D, E, F, G, H, I, J, K	11
Noonan et al (2020)	Cohort	A, B, C, D, E, F, G, H, I, J, K	11
*Oberoi et al (2017A)	Longitudinal	A, B, C, D, E, F, G, H, I, J, K	11
*Oberoi et al (2017B)	Longitudinal	A, B, C, D, E, F, G, H, I, J, K	11
*Oberoi et al (2017C)	Longitudinal	A, B, C, D, E, F, G, H, I, J, K	11
Oerlemans et al (2012)	Longitudinal	A, B, C, D, E, F, G, H, I, J, K	11
Parry et al (2012)	Cross‐sectional	A, B, C, D, E, H, I, J, K	9
Parry et al (2011)	Semi‐structured interviews	A, B, C, D, E, F, G, H, I, J, K	11
Paul et al (2017)	Cross‐sectional	A, B, D, E, F, G, H, I, J, K	10
Posluszy et al (2016)	Cross‐sectional	A, B, C, D, E, F, G, H, I, J, K	11
Raphael et al (2017)	Semi‐structured interviews	A, B, C, D, E, F, G, H, I, J, K	11
Smith et al (2010)	Cross‐sectional	A, B, C, D, E, F, G, H, I, J, K	11
Smith et al (2009)	Cross‐sectional	A, B, C, D, E, F, G, H, I, J, K	11
Swash et al (2018)	Focus groups (three)	A, B, D, E, F, G, H, I J, K	10
Tzelepis et al (2018)	Cross‐sectional	A, B, C, D, E, F, G, H, I, J, K	11
Vargas‐Roman et al (2020)	Systematic review & meta‐analysis	A, B, C, D, E, F, G, H, I, J, K	11
Vena et al (2021)	Meta‐ethnography	A, B, C, D, E, F, G, H, I, J, K	11
Xu et al (2020)	Cross‐sectional	A, B, C, D, E, F, G, H, I, J, K	11
Zucchetti et al (2017)	Cross‐sectional	A, B, C, D, E, F, G, H, J, K	10

^*^ Merged datasets.

**TABLE A4:** Study characteristics (*n* = 47)

Quantitative							
Reference	Sample size	Aim	%	%	Age range (mean) in years	Mean time since diagnosis (SD)	Key findings
			Male	Lymphoma			
*Country*	*Recruitment*						
**Arboe 2017**	**369**	Describe the social outcomes after ASCT in terms of return to work.	60%	100% NHL	22–73 (median 58, SD NR)	NR	⁃ Following ASCT, there was an impaired association between return to work for patients on sick leave at the time of relapse.
*Denmark*	*National registry*						
							⁃ Older patients (>55 years) were more likely to return to work compared to younger patients.
**Arden‐Close 2011**	**115**	Evaluate gender differences in HRQoL, late effects, perceived vulnerability, and satisfaction with care.	49%	100%	18–45 (mean 37.3, SD 5.6)	12.8 years (SD 5.0)	⁃ No gender differences were found in self‐reported late effects or perceived vulnerability, however, men with more late effects reported worse psychological HRQoL.
*United Kingdom*	*Single site*						
							⁃ Men wished to talk about more topics than they did, while women were able to talk about the topics they wanted.
**Beaven 2016**	**553**	Evaluate differences in QoL between incurable indolent lymphoma and potentially cured aggressive NHL.	50%	100% NHL	(Mean 61.9, SD 13.5)	10.6 years (SD 6.9)	⁃ Aggressive and indolent lymphoma survivors were found to have similar overall QoL, however differences were greater in short‐term survivors.
*United States*	*Two sites*						⁃ Longer‐term indolent lymphoma survivors had higher QoL scores than shorter‐term survivors.
**Behringer 2016**	**3759 (5 years follow up n = 1758)**	Investigate the persistence of severe fatigue in HL survivors up to 9 years after primary therapy and its relationship with social reintegration.	56%	100% HL	18–60 (median 34)	Unclear	⁃ Baseline severe fatigue was often younger, more often female, and had a higher stage of disease compared to patients without a baseline of severe cancer‐related fatigue.
							⁃ Five years post‐therapy, 84% of survivors without severe fatigue were working or in education, compared to 57% of survivors' experiencing severe fatigue.
*Germany*	*Database*						
**Chen 2012**	**511**	Evaluate the disparities in employment and insurance between HL survivors and their non‐diagnosed peers.	49.5%	100% HL	16–82 (median 44)	Median 15 years (range 5–32)	⁃ HL survivors were more likely to report job denial, difficulty obtaining insurance due to medical history, and difficulty changing jobs due to fear of losing insurance.
*United States*	*Single site*						
							⁃ Male gender, income, and scarring of the head and neck were associated with job denial (multivariate analysis).
**Esser 2018**	**922**	Compare adjusted levels of QoL across different subsamples of survivors with haematological malignancies versus the general population.	57% (total sample)	57%	(Mean 63.9, SD 13.4)	9.1 years (SD 4.2)	⁃ Worse QoL predictors included: being female, not being in remission at the time of the survey, number of comorbidities, and having a history of relapse.
*Germany*	*Two cancer registries*						⁃ Compared with the general population, haematology cancer survivors scored significantly lower in functioning and higher in symptom burden.
**Fauer 2021**	**1151**	Examine factors influencing patient experiences in care during the first year of diagnosis in older adults diagnosed with leukaemia and lymphoma.	46% (lymphoma sample)	63%	65–85+	Median 6 months	⁃ Completing the survey 8–12 months after diagnosis compared to 0–3 months was associated with a higher global rating of care (fully adjusted models).
							⁃ Each additional comorbidity (β 0.26, *p* = 0.003) and being dually eligible for Medicaid (β 0.43, *p* = 0.03) were significantly associated with a higher adjusted personal doctor rating.
*United States*	*Database (NCI SEER)*						
**Georges 2020**	**389**	Explore the late effects and QoL in long‐term survivors after autologous haematopoietic cell transplantation.	59% (lymphoma sample)	69%	22–88 (median 63)	NR	⁃ Lymphoma survivors were more likely to report post‐traumatic stress symptoms (6% vs. 1%) and problems with sexual desire, erection, ejaculation, or vaginal dryness or pain (62% vs. 51%) compared to myeloma survivors.
*United States*	*Single site*						
							⁃ Worse mental functioning was associated with younger age and treatment for anxiety, depression, or pain.
**Greaves 2014**	**718**	Determine the impact of haematological malignancy on fertility and sexual function based on the patient's report of their experience.	59% (total sample)	84%	(Mean HL 53.1, SD 11.9; NHL 45.0, SD 13.0)	HL 25.1 years (SD 10.0); NHL 16.8 (SD 8.5)	⁃ Fertility support services were attended by few (12%).
							⁃ Of the HL patients who did not store a sperm sample, the majority (52%) reported that they were not offered the chance to store a sperm sample, whereas among NHL patients most (55%) of those who did not store a sperm sample did not intend to have any children after treatment.
*United Kingdom*	*Single site*						
**Hall 2015** **^a^**	**715**	Identify the most prevalent unmet needs of haematological cancer survivors.	59% (total sample)	64%	15–80	35 months (SD 18.5)	⁃ ‘Dealing with feeling tired’ (17%), was the most frequently endorsed ‘high/very high’ unmet need.
							⁃ Higher levels of psychological distress (e.g., anxiety, depression, and stress) and indicators of financial burden because of cancer were consistently identified as characteristics associated with the three most prevalent ‘high/very high’ unmet needs.
*Australia*	*Four cancer registries*						
**Hall 2014** **^a^**	**696**	Identify subgroups of haematological cancer survivors who report a high level of multiple unmet needs.	59% (total sample)	64%	15–70+	1–60+ months	⁃ ‘High/very high’ level of unmet need on seven or more items of the SUNS were reported by 25% of participants.
							⁃ Survivors who: had relocated due to their cancer, had difficulty paying bills, had used up their savings because of cancer, and were classified as having above‐normal symptoms of depression and stress had statistically significantly higher odds of reporting seven or more ‘high/very high’ unmet needs.
*Australia*	*Four cancer registries*						
**Hall 2013**	**437**	Assess and compare the unmet needs of Australian (A) and Canadian (C) haematological cancer survivors.	59% (A); 54% (C)	>50% (A); >52% (C)	15–60+	1–60 months	⁃ ‘Dealing with feeling tired’ was identified as the highest concern by survivors.
							⁃ Having a personal expense in the last month because of having cancer, younger age at diagnosis, female sex, vocational or other level education, and consulting a health care professional for cancer treatment or concerns about cancer in the last month were associated with multiple areas of need.
*Australia*	*Two national registries*						
**Hernaes 2021**	**225**	Investigate post‐treatment work patterns amongst survivors of lymphoma treated with high‐dose chemotherapy and ASCT.	61%	100%	(Mean 52, SD 11.6)	NR	⁃ Eighty‐five per cent of participants were employed when diagnosed, 77% before high‐dose chemotherapy and autologous stem‐cell transplantation and 69% at the survey.
*Norway*	*Hospital registry*						
							⁃ Employment before HDT‐ASCT positively corresponds with a higher probability of employment at survey for a given symptom burden.
**Husson 2017**	**198**	Examine differences in HRQoL between AYA lymphoma survivors and a normative population and to determine sociodemographic, clinical, and long‐term symptom‐related factors associated with HRQoL.	57%	100%	(Mean 34.7, SD 7.4)	4.2 years (SD 2.7)	⁃ Poorer HRQoL for AYA lymphoma survivors was observed in seven domains: physical, role, cognitive, emotional, social functioning, fatigue, and financial difficulties compared to the normative population sample.
*The Netherlands*	*National survivorship registry*						⁃ Being unemployed, female gender, having one or more comorbid conditions, high levels of fatigue and psychological distress were most strongly associated with HRQoL.
**Kang 2018**	**370**	Compare HRQoL at diagnosis to that of long‐term follow‐up among survivors of aggressive and indolent NHL.	55.5%	100% NHL	18–82 (median 51)	Median 4.0 years (range 1.7–17.4 years)	⁃ The HRQOL of long‐term survivors with aggressive NHL improved to a similar level of indolent NHL during the follow‐up survey. However, survivors of NHL were found to fear the probability of relapse and second malignancy.
*South Korea*	*Single site*						⁃ More than 65% of survivors thought they did not receive sufficient support from others, and patients who had financial difficulties at diagnosis were more frequently associated with suffering from insufficient support.
**Keegan 2012**	**523**	Describe unmet information and service needs of AYA survivors and identify sociodemographic and health‐related factors associated with unmet information and service needs.	63% (total sample)	52%	15–39	Median 11 months	⁃ Half of survivors have unmet information needs (about cancer treatments, long‐term effects, cancer returning or new cancer type).
*United States*	*Multiple registries*						
							⁃ Unmet needs included: staying physically fit (30%), financial support (>50%), fertility or reproductive concerns about having children in the future (>40%).
**Khimani 2013**	**273**	Determine changes in QoL and the development of new late effects over seven years in a cohort of long‐term HL survivors.	50%	100%	31–78	NR	⁃ Over 7 years, a significant physical decline was noted, decline was greater among survivors experiencing new cardiac or pulmonary complications compared with those without any new complications.
*United States*	*Not clear*						⁃ There were no differences in changes in QoL scores over time between survivors who did or did not develop a new infectious complication or a new second malignancy.
**Kim 2017**	**826**	Describe the prevalence and correlates of unmet needs among lymphoma survivors and to identify their association with HRQoL.	58%	100% NHL	(Mean 56, SD 12.0)	6.3 years (SD 3.2)	⁃ The most reported unmet needs domains: ‘treatment and prognosis’ (38.3%) and ‘keeping mind under control’ (30.5%).
							⁃ The three most often reported individual unmet needs: ‘being informed about prevention of recurrence’ (50.7%), ‘being informed about prevention of metastasis’ (49.7%), and ‘having self‐confidence of overcoming cancer’ (42.7%).
*South Korea*	*Three hospital registries*						
**Kim 2014**	**837**	Examine the association between HRQOL and sociodemographic and clinical factors and identify predictors of HRQoL in the survivor population.	57%	100%	(Mean 54.6, SD 12.6)	6.3 years (SD 3.2)	⁃ Overall, the HRQoL in both HL and NHL survivors and the general population were comparable, but clinically meaningful worse social functioning in NHL survivors (*p* < 0.001) and more severe fatigue in HL survivors (*p* < 0.001) than in the general population was observed.
*South Korea*	*Three hospital registries*						
							⁃ Survivors who received peripheral blood stem cell transplants showed clinically meaningful worse role (*p* = 0.001) and social (*p* < 0.001) functioning than those who were treated with first‐line chemotherapy alone.
**Kreissl 2020**	**4215**	Analyse longitudinal HRQoL data prospectively collected within trials.	55%	100%	18–60 (median 34)	0–5 years	⁃ During survivorship, cognitive, emotional, role, and social functioning and fatigue, dyspnoea, sleep, and financial problems were severely and persistently affected.
*Germany*	*Pooled secondary analysis*						
							⁃ Financial problems were the most affected domain of HRQoL in the first year after treatment.
**Lekdamrongkul 2021**	**312**	Exploring HRQoL among non‐Hodgkin's lymphoma survivors after completion of primary treatment.	45%	100% NHL	18–89 (mean 58, SD 14.67)	NR	⁃ NHL survivors in phase I (<6 months post‐treatment) had significantly lower physical well‐being and functional well‐being scores than longer‐term survivors; and significantly lower lymphoma domain scores than those in phase III (>4 – 9 years after completion of primary treatment).
*Thailand*	*Two sites*						
							⁃ Physical symptom distress, anxiety, depression, unmet supportive care needs, poor adaptation, and receiving chemotherapy disrupted HRQoL (all *p* < 0.001).
**Lobb 2009**	**66**	Determine patients' information, emotional and support needs after treatment for haematological malignancy.	NR	59%	24–82 (mean 54, SD 14.07)	NR	⁃ The most often endorsed unmet needs included: managing the fear of recurrence, the need for a case manager and the need for communication between treating doctors.
*Australia*	*Two sites*						⁃ Predictors of unmet needs included younger patients (*p* = 0.01), marital status (*p* = 0.03) and employment (*p* = 0.03).
**Ng 2016**	**156**	Determine the prevalence of anxiety and depression of lymphoma survivors and to investigate associations between these disorders and QoL.	40%	100%	18–85 (median 52.06, SD 16.8)	6.82 years (SD 5.5), median 26 (range 1–27)	⁃ 18% of patients had symptoms of anxiety and 10% had symptoms of depression.
							⁃ Patients with anxiety were associated with lower overall QOL score, lower emotional and cognitive functioning and complained more of fatigue and insomnia (*p* < 0.05). Patients who had depression were associated with lower physical functioning and complained more of insomnia (*p* < 0.05).
*Malaysia*	*Single site*						
**Noonan 2020**	**566**	Evaluate QoL and impact of cancer difference in rural and non‐rural non‐Hodgkin lymphoma survivors.	48%	100% NHL	23–65+	15.2 years (SD 7.19)	⁃ Rural residence was independently associated with lower SF‐36 physical part summary scores and the physical function subscale (all *p* < 0.05).
*United States*	*Pooled secondary analysis*						
							⁃ Rural residence was also associated with higher IOCv2 positive impact scores and the subscales of altruism/empathy and meaning of cancer scores in the adjusted models (all *p* < 0.05).
**Oberoi 2017A** **^a^**	**414**	Examine the influence of anxiety, depression, and unmet supportive care needs on future QoL in MM and DLBCL.	57% (total sample)	57% NHL (DLBCL)	(Mean 63.82, SD 11.08)	6.7 months (SD 1.98)	⁃ Except for physical well‐being, all other QoL subscales and overall QoL were significantly associated with T1 (on average 7 months post‐diagnosis) anxiety.
*Australia*	*Cancer registry*						⁃ All QoL subscales and overall QoL were significantly associated with T1 depression. Only patient care needs were associated with physical and social well‐being and overall QoL.
**Oberoi 2017B** **^a^**	**414**	Examine the cross‐sectional and longitudinal associations between patient‐reported unmet needs and anxiety and depression for survivors.	57% (total sample)	57% NHL (DLBCL)	(Mean 63.82, SD 11.08)	6.7 months (SD 1.98)	⁃ At T1 (on average 7 months post‐diagnosis), 30% of participants reported at least one moderate to high unmet need in the psychological needs and the physical and daily living needs domains, 24% information needs, 9% sexuality needs and 11% patient care needs.
*Australia*	*Cancer registry*						
							⁃ At T2 (on average 15 months post‐diagnosis), 23% reported at least one moderate to high unmet need in the psychological and physical and daily living needs domains, 14% information needs and 8% sexuality and patient care needs.
**Oberoi 2017C** **^a^**	**414**	Examine the course of anxiety, depression, and unmet needs in survivors in the first 2 years post‐diagnosis.	57% (total sample)	57% NHL (DLBCL)	(Mean 63.82, SD 11.08)	6.7 months (SD 1.98)	⁃ Course of anxiety differed (*p* < 0.01) with the rate increasing in DLBCL (14%–22%) while staying stable for MM (15% to 12%).
*Australia*	*Cancer registry*						⁃ Change in unmet needs was similar for the two cancer groups, except for moderate to high psychological needs (*p* < 0.05).
**Oerlemans 2012**	**1135**	Measure the perceived level of satisfaction with information received by survivors of lymphoma and MM.	60% (total sample)	86%	(Mean 61.6, SD 14)	3.7 years (SD 2.7)	⁃ Two‐thirds of survivors were satisfied with the amount of received information, with HL survivors being most satisfied (74%). At least 25% of survivors wanted more information.
							⁃ Younger age, having had chemotherapy, having been diagnosed more recently, using the Internet for information, and having no comorbidities were key factors associated with higher perceived levels of information provision.
*The Netherlands*	*National registry*						
**Parry 2012**	**477**	Characterise the psychosocial and health service needs of adult leukaemia and lymphoma survivors after treatment.	54% (total sample)	79%	18–85 (mean 56, SD NR)	NR	⁃ The rate of unmet needs was highest for sexual issues, handling medical and living expenses, emotional difficulties, employment, and health insurance.
							⁃ Women were more likely to report unmet childcare needs than men; younger individuals were more likely to report needing help with emotional difficulties and family problems, and lower income was related to greater unmet needs for medical and living expenses.
*United States*	*Cancer registry*						
**Paul 2017**	**787**	Explore the dyadic relationships between unmet needs, depression, and anxiety in people diagnosed with haematological cancer and their support persons.	58% (total sample)	56% NHL	(Mean 57, SD 13)	NR	⁃ Survivor unmet needs were significantly related to support person depression (*p* = 0.0036).
							⁃ Survivor unmet needs did not have a significant relationship to supporting person anxiety (*p* = 0.78). Higher anxiety scores for survivors were significantly associated with greater support person's unmet needs.
*Australia*	*Five state cancer registries*						
**Posluszy 2016**	**429**	Examine the existential challenges that lymphoma cancer survivors may experience.	44%	100%	18–78 (mean 44.2, SD 12.7)	7 years (SD 7.4)	⁃ Most survivors (73–86%) endorsed existential concerns, with 30−39% reporting related perceived functional impairment.
*United States*	*Online survey*						⁃Concerns were associated with being female, younger, unmarried, and having undergone stem cell transplantation.
**Smith 2010**	**652**	Examine the association between the impact of cancer and QoL post‐treatment for NHL survivors.	50%	100% NHL	(51.9, SD 14.2)	10.8 years (SD 7.5)	⁃ Survivors with comorbidities and negative appraisals of life threat and treatment intensity reported worse physical and mental health and QOL (all *p* < 0.05).
*United States*	*Two cancer registries*						
							⁃ After controlling for demographic and clinical characteristics, younger respondents reported better physical but worse mental health and QoL (all *p* < 0.01).
**Smith 2009**	**761**	Compare the QoL status of individuals with active NHL with those who were disease‐free.	50%	100% NHL	25–92 (mean 52.3, SD 14.1)	10.4 years (SD 7.2)	⁃ Survivors with active disease (*n* = 109) demonstrated worse physical and mental health functioning, worse QOL, and less positive and more negative impacts of cancer compared with disease‐free survivors (*n* = 652; all *p* < 0.01).
*United States*	*Two cancer registries*						
							⁃ No significant differences were seen between short‐term survivors (2 – 4 years post‐diagnosis) and long‐term survivors (>5 years post‐diagnosis).
**Tzelepis 2018**	**1511 (Urban n = 1145,** **Rural n = 272)**	Examine the unmet supportive care needs among rural versus urban haematological cancer survivors.	57% (total sample)	67%	(Mean 58, SD 13)	3.4 years (SD 1.5)	⁃ Dealing with feeling tired was the most common ‘high/very high’ unmet need for rural (15%) and urban (15%) survivors. The emotional health domain had the highest mean unmet need score for rural and urban survivors.
							⁃ Rurality was associated with a decreased unmet emotional health domain score while travelling for more than 1 h to treatment was associated with increased unmet financial concerns and unmet access and continuity of care.
*Australia*	*Five state cancer registries*						
**Xu 2020**	**1549**	Assess the association of HRQoL with financial burden among patients with NHL in China.	52%	100% NHL	(Mean 43.3, SD NR)	Not clear	⁃ Sixty per cent of respondents reported suffering moderate to high financial burdens.
							⁃ A significant relationship between increased financial burden and reduced HRQoL scores, including the EQ‐Index, physical, emotional, and social functioning, was found.
*China*	*Online survey*						
**Zuchchetti 2017**	**50**	Investigate the experience of possible body image discomfort among AYA haematological cancer survivors.	52% (total sample)	52%	15–23 (mean 17.7, SD 2.53)	NR	⁃ No significant differences in body image were found between leukaemia and lymphoma survivors or between the off‐therapy and long‐term groups.
*Italy*	*Single site*						
							⁃ More females were in the risk category of impaired body image than males.
**Qualitative**							
**Chen 2020**	**12**	Determine the perceived unmet needs regarding lymphoma care in rural areas.	50%	100%	21–73 (median 54)	NR	⁃ The greatest barrier to care was the travel distance. The participants described difficulty navigating between local clinics and larger cancer centres.
							⁃ The lack of communication between the local and specialised clinics complicated the process, and participants had difficulty contacting or seeking advice from the team at the larger cancer centres.
*United States*	*Single site and via conferences*						
**Hackett 2018**	**14**	Explore lymphoma survivors' experiences on their end of treatment and follow‐up care.	64%	100%	18–65+	3–60 months	⁃ Themes found: (i) dealing with uncertainty, (ii) changing relationships, (iii) returning to work, (iv) extended recovery time and (v) concerns for the future.
							⁃ Some participants were unaware that their treatment had ended, many experienced recurrent infections which prolonged recovery time, and many had no recall of discussions on healthy lifestyle behaviours or recommended screening programmes at their follow‐up visits.
*Ireland*	*Single site*						
**Herrmann 2020**	**17**	Explore the unmet needs experienced by haematological cancer survivors as a result of their disease and treatment and helpful strategies.	59% (total sample)	71%	19–76 (mean 57, SD 13)	0–2+ years	⁃ Themes found: (i) changes in unmet needs across the care trajectory; (ii) informational unmet needs requiring improved patient‐centred communication; (iii) uncertainty about treatment and the future; (iv) coordinated, tailored, and documented post‐treatment care planning as a strategy for optimal care delivery; and (v) ongoing support services to meet psychosocial and practical unmet needs.
*Australia*	*One state cancer registry*						
**Monterosso 2017**	**17**	Explore the post‐treatment experiences and preferences for follow‐up support of lymphoma survivors.	53%	100%	27–85 (mean 63.8, SD 14.5)	6–29 months	⁃ Themes found: (i) information; (ii) loss and uncertainty; (iii) family, support and post‐treatment experience; (iv) transition, connectivity and normalcy, and (v) person‐centred post‐treatment care.
*Australia*	*Single site*						
							⁃ Participants described a sense of loss as they transitioned away from regular interaction with the hospital at the end of treatment, but also talked about the need to find a ‘new normal’.
**Murphy‐Banks 2022**	**17**	Understand how survivors in active survivorship care perceived their role in treatment decision‐making and when they acquired an understanding of late effects.	47%	100% HL	18–39+	5–36 years (mean 19 years)	⁃ Role in initial treatment decision‐making fluctuated between passive and active engagement with providers identified as being crucial to this process.
							⁃ Half of the interviewees (53%) expressed unmet information needs. Most participants (71%) reflected on fertility discussions; more than half of those participants cited fertility discussions occurring primarily before or during treatment.
*United States*	*Not clear*						
**Parry 2011**	**51**	Explore patient's experiences on care delivery during the end‐of‐treatment transition to survivorship for leukaemia and lymphoma survivors.	45% (total sample)	76%	20–82 (Mean 50.3, SD NR)	Not clear	⁃ Survivors reported poor continuity of care across the patient–survivor transition, difficulty finding proper information/services, lack of preparation, lack of support for survivorship issues, and inadequate or poorly timed follow‐up as factors contributing to adjustment difficulties at end of treatment and beyond.
*United States*	*Cancer support organisations*						
**Raphael 2017**	**23**	Explore the nature and timing of psychosocial distress experienced by haematological cancer survivors.	44% (total sample)	74%	33–77	0–8 years	⁃ Themes found: (i) apprehension about leaving the safety of the health care system (ii) uncertainty and life transitions in the post‐treatment period (iii) distress associated with ongoing physical problems or impairment, and (iv) fear of recurrence.
*New Zealand*	*National registry*						
**Swash 2018**	**6**	Investigate the experiences of psychosocial needs in haematological cancer patients, the importance, and impact of their unmet needs.	83%	100% NHL	NR	NR	⁃ Themes found: (i) concerns for family, (ii) information needs, and (iii) the need for psychological support.
							NHL patients perceive themselves as different from other cancer survivors or patients (i.e., treated by haematologists rather than oncologists; lymphoma can be described as a chronic health condition, rather than acute cancer).
*United Kingdom*	*Single site*						

Reviews				
Reference	Country	No. of Articles	Aim	Key findings
**Arden‐Close 2010**	*United Kingdom*	18	Determine HRQoL in survivors of HL and NHL.	⁃ Survivors of lymphoma experienced worse physical but comparable mental HRQoL to the general population.
				⁃ No conclusions could be drawn about the association between different treatments and HRQoL.
**Vargas‐Roman 2020**	*Spain*	15	Examine the prevalence of anxiety among lymphoma patients.	⁃ The meta‐analysis sample was *n* = 2138 and the overall prevalence of anxiety in lymphoma patients was 19% (95% CI [12%, 25%]).
**Vena 2021**	*United States*	9	Appraise the experiences of adults with lymphoma at the acute and chronic survivorship phases.	⁃ Themes found: (i) the challenges and burden of treatment, (ii) communication and support from others, and (iii) moving forward with life.

*Note*: Bold was used visually to enhance visability of author and sample size.

Abbreviations: AL, Acute Leukaemia; AYA, Adolescent and Young Adult; CI, Confidence Interval; DLBCL, diffused large b‐cell lymphoma; HDT‐ASCT, high dose chemotherapy and autologous stem cell transplantation; HL, Hodgkin Lymphoma; HRQoL, Health‐Related Quality of Life; IOCv2, Impact of Cancer version 2; MM, multiple myeloma; NCI SEER, National, Cancer Institute (NCI) Surveillance; NHL, non‐Hodgkin Lymphoma; NR, not reported; QoL, Quality of Life; SD, standard deviation; SF‐36, Medical Outcomes Study 36‐item Short Form Survey.

^a^ Merged databases.

### 3.4 Disparity in health service delivery

The uncertainty and life transitions in the post‐treatment period included what to do now or how to return to normal and receiving guidance or information relevant to this was essential to survivors.^47^, ^48^, ^61^, ^62^, ^63^ The transition away from the safety net of hospital interaction at the end of treatment was described as a sense of loss or abandonment by the health system, while others discussed the need for a new normal.^47^, ^62^ Transitions to follow‐up care in fragmented health service systems were reported.^5^ While survivors felt a need to make sense of the cancer experience, some reported seeing others ‘survive and thrive’ after cancer as empowering^60^ pp. 1470.

A lack of targeted and speciality services for lymphoma contributed to survivor distress.^5^, ^47^ For lymphoma survivors, limited financial and psychosocial support was available compared to other cancer groups such as breast cancer.^47^ From a US perspective, speciality lymphoma care providers in smaller hometowns were scarce and contributed to negative experiences of diagnosis, including, in some cases, incorrect diagnosis.^5^ In addition, travel distance was a barrier to healthcare for some survivors with long commutes and travel costs.^5^, ^48^ The diagnosis of haematological malignancy in older persons is frequently delayed due to issues with referrals, general practitioners' limited knowledge of haematological pathologies and complications of comorbidities in masking symptoms.^35^ Qualitative reports from participants show an acute awareness of the busy clinical environment and demands on healthcare professionals; this was a barrier to some survivors expressing their needs with healthcare professionals as they did not feel comfortable disturbing staff.^60^

### 3.5 The psychological impact of cancer

The fear of cancer recurrence was a predominant concern reported by lymphoma survivors.^40^, ^59^, ^61^, ^62^, ^63^, ^64^ For some, any physical symptom experienced instilled fear of recurrence between follow‐up appointments. A heightened fear was experienced before and during scheduled appointments.^61^ Help to manage the fear of recurrence was a frequently endorsed need in reviewed studies.^40^, ^63^, ^64^ The fear of recurrence was described as a shared experience of living with uncertainty and fear for some focus group participants (*n* = 17).^62^ However, the degree of fear of recurrence was not different between stages of aggressive NHL.^59^ Other means of psychological distress were reported by many lymphoma survivors, including depression, anxiety, and stress.^3^, ^21^, ^41^, ^42^, ^45^, ^63^, ^65^, ^66^, ^67^ Participants with above typical symptoms of depression and stress had statistically significant odds of reporting multiple high‐level unmet needs.^21^, ^39^

Younger participants reported better physical but worse mental health.^68^ The initial delivery of diagnosis required a psychological adjustment that was considered more complex than the physical impact of the disease.^48^, ^60^ While some survivors felt their perceived need for help peaked just after being diagnosed, for others, their need for help escalated during cancer recurrence.^48^ One study reported the psychosocial impact of body image for this cancer group; female survivors' body image is more impaired and markedly varies from their male counterparts, with more concern relating to their physical appearance than males.^33^ Furthermore, an interesting finding from focus groups in the UK was how survivors felt different to other cancer patients.^60^

Many lymphoma survivors reported impaired neurocognitive functioning affecting younger survivors of both NHL and HL.^34^, ^55^, ^59^, ^69^ Impaired cognitive functioning at diagnosis for NHL survivors was significantly associated with a lack of life purpose in long‐term survivors.^59^ Almost one‐third of participants (*n* = 523) in a cross‐sectional survey indicated a need to avail psychological health care. More than half stated this need was left unmet.^52^ Over 40 per cent of NHL survivors (*n* = 370) from South Korea reported they were not happy or satisfied with their life.^59^ While a US cross‐sectional survey study found NHL survivors with active disease (*n* = 109) had more negative impacts of cancer compared with disease‐free survivors (*n* = 652).^56^

### 3.6 Impactful and debilitating concerns

A substantial burden of cancer and its treatment has been reported by lymphoma survivors, including many who are free of disease.^61^ Over half of the survivors developed one or more late effects in a longitudinal study conducted over seven years, cardiac complications being the most common.^26^ Other physiological issues identified included loss of energy, loss of strength, recurrent infections, neurocognitive decline, neuropathy, lymphadenopathy, pruritis, weight changes and secondary cancers.^56^, ^61^, ^62^, ^69^, ^70^ While this reflects the wide range of problems affecting lymphoma survivors, it is important to note the significant heterogeneity in reporting what constitutes ‘common’ symptoms for this cohort. Worse physical health and worse fatigue compared to the general population were found in lymphoma survivors.^55^, ^57^, ^69^ Some survivors had physical complaints which impacted their daily lives; others felt an indescribable change in how they physically felt.^63^ Survivors with active disease, long‐term disease, comorbidities, of older age or rural residence were associated with worse physical functioning.^56^, ^68^, ^69^, ^71^

Cancer‐related fatigue persistently affects individuals with lymphoma and impacts quality of life.^39^, ^55^, ^72^ General practitioner visits were higher (almost double) for participants experiencing severe cancer‐related fatigue than those without severe fatigue.^72^ Prevalence of severe cancer‐related fatigue in HL survivors was reported to reduce over 5 years; however, a limitation of this study is that the sample also reduced over this time (baseline *n* = 3759; 5 years follow‐up *n* = 1758).^72^ Severe fatigue was a barrier to social reintegration and impacted survivors' capacity for social events, work commitments or even family time.^61^, ^72^ Two studies compared groups of survivors by residence, rural versus urban and by country Australian survivors with Canadian survivors; both reported dealing with feeling tired was their most commonly reported unmet need.^38^

Given the mean age of most survivor samples was greater than 40 years of age, fertility concerns were scarcely reported. Infertility concerns, including the likelihood of remaining childless compared to a normative control population, were discussed.^37^, ^61^ Two studies discussed the use of sperm banking by male participants before treatment,^37^, ^53^ while no studies reported its gender counterpart of egg harvesting. Sexual issues were among the highest‐rated unmet needs for one study,^44^ with sexual changes mentioned by others.^37^ A negative impact on sexual function resulting from cancer was seen more often in women or survivors using anti‐depressants or experiencing body image concerns.^37^

### 3.7 The monetary cost of survival

Unmet financial and work‐related needs were reported by many lymphoma survivors.^21^, ^34^, ^39^, ^44^, ^49^, ^50^, ^52^, ^55^, ^57^, ^59^, ^73^ Survivors worried about earning money, having trouble meeting daily expenses, relocating due to cancer caused difficulty paying bills and depleted savings and were associated with unmet needs.^21^, ^39^ Over 65 per cent of survivors (*n* = 370) of a single centre cohort study reported not receiving enough support from others, with financial difficulties more frequently associated with insufficient support.^59^

Differences in financial needs related to care delivery internationally were evident. Participants in a US study reported a lack of transparency from the billing department at a university hospital. A survey conducted in China recommended that medical professionals select cost‐effective treatments while ensuring the patients understand the financial consequences of treatments.^5^, ^74^ Conversely, in Denmark, individuals were financially supported through state benefits.^54^ This highlights substantial discrepancies in the financial implications for lymphoma survivors based on their country of residence due to the availability or lack of financial support systems relating to lymphoma care.

From Germany to China, a large percentage (40% – 85%) of lymphoma survivors from multiple countries reported being members of the workforce. Staying employed from diagnosis throughout treatment is associated with a consistently higher likelihood of employment later in life, regardless of symptom burden.^73^ Working gave some survivors a sense of purpose and financial security, while flexible working arrangements helped meet psychological and practical needs.^48^ Sometimes, survivors experienced cost implications when adjustments to work or school were made due to cancer care.^53^ Similarly, patients on sick leave during relapse following autologous stem cell transplantation were found to have a poorer prognosis relating to returning to work and a higher rate of disability pension.^54^ Lower income was associated with more significant unmet needs relating to expenses for medical and living costs.^44^ Furthermore, lymphoma survivors may be immunocompromised, which can be a substantial barrier to returning to work.^61^ However, for some survivors, a return to work was seen as a return to normal.^61^

### 3.8 Insufficient provision of survivorship information

Unmet information needs were prevalent in lymphoma survivors from varying countries and research approaches.^43^, ^47^, ^48^, ^51^, ^52^, ^58^, ^60^, ^62^ A lack of sufficient information that was helpful for patients in understanding their care was reported by several survivors of one study (*n* = 17).^48^ Receiving information relating to survivorship, such as possible long term side effects of treatment, being informed about prevention of recurrence or recommended screening programmes, were reported by few.^52^, ^58^, ^61^ While explicit conversations about late effects were desired by all participants of semi‐structured interviews (*n* = 17).^53^

Monterosso, Taylor^62^ suggested some health care professionals did not anticipate the required information and support needed post‐treatment leaving survivors' information needs unmet. While survivors from another study felt health care providers were unprepared to help them with survivorship concerns, including physical and psychosocial issues in the posttreatment period.^47^ The delivery and the personalisation of information relevant to the individual were considered important.^60^ Moreover, comprehensive verbal information supplemented with written take‐home information was appreciated by survivors.^48^

There were gender differences in the provision of information needs, with males being more likely to report unmet information needs.^52^ Men wanted to discuss more than they did, while women managed to discuss the topics they wished to discuss.^51^ However, one study found that satisfaction with information provision was relatively good in two‐thirds of survivors, with HL the most satisfied (74%).^43^ The use of technology was seen as favourable for improved communication with clinical teams by survivors.^5^ Similarly, Survivorship Care Plans may provide opportunities for good information provision regarding the ongoing sequelae of survivorship concerns between healthcare professionals and survivors.^43^

## 4 DISCUSSION

Research surrounding the needs of adult lymphoma survivors is emerging and increasing. Despite the advancement and positive associations with lymphoma survivorship, it is not without consequence, as survivors are living with and beyond a lymphoma diagnosis with a myriad of needs and compromised quality of life.

The disparity of health service delivery for survivors resulted in individualised responses to the transition after treatment from a sense of abandonment to a welcomed change. Many lymphoma survivors experienced a prominent need for help with the psychological impact of cancer. Fear of recurrence was a common concern with depression, anxiety, stress, body image and neurocognitive functioning affecting the psychological needs of lymphoma survivors. Varying symptom burdens like physiological concerns or late effects highlight the heterogeneity among lymphoma survivors. However, cancer‐related fatigue and feeling tired were debilitating issues and the most prevalent unmet need for some. Unmet needs associated with finances and work were a prominent concern relating to worries about money and barriers to employment because of a lymphoma diagnosis. Similarly, unmet information needs were prevalent with the provision of survivorship information, such as late effects left desired.

Limited evidence suggests health service delivery is fragmented for lymphoma survivors with a lack of available speciality services. Some survivors felt lymphoma was different to other cancers, while others noted more supports were available for breast cancer as opposed to lymphoma. Moreover, lymphoma diagnosis can often be delayed, negatively impacting patient outcomes. While evidence on the factors influencing this is limited, they may include the complexity of older age and delayed recognition by non‐haematological speciality health providers such as general practitioners.^8^, ^35^ Yet it is vital to acknowledge the complexities of these issues, which may be multifactorial, involving both patient and health service provider‐related problems and solutions.^9^

Similar to a systematic review conducted on factors associated with fear of recurrence in cancer patients, with over half of included studies focused on breast cancer, there was an association between physical symptoms for lymphoma survivors and the fear of cancer recurrence.^75^ Financial hardship among survivors has been well documented; however, substantial heterogeneity in its prevalence was found by a systematic review of mixed cancer types.^76^ For lymphoma, this rapid review found that many survivors are among our workforce internationally, and financial and work‐related concerns are commonly associated with unmet needs. Of the included studies reporting participants' employment status, a sizeable percentage (40% – 85%) of lymphoma survivors are in the global workforce. Therefore, enhanced awareness of the occupational wellbeing of lymphoma survivors is required of employers, colleagues and insurance companies as the effects of cancer and its treatment can linger long past treatment completion.^77^

Additionally, there is a need for better information provision for patients on the cost of ongoing treatment relative to quality of life. Mitigating against the delivery of toxicities with potentially little gain to survival, especially for elderly populations.^78^, ^79^ More research into the challenging concern of toxicity versus quality of life in this population is needed to support healthcare professionals and health systems. Fertility is one problem specific to younger lymphoma survivors. Survivors may feel uncertain about their fertility post‐treatment and available preservation strategies (i.e., egg harvesting, sperm banking).^80^ Being cognisant of survivors' preferences is essential in developing novel survivorship services to optimise engagement.^81^ However, the current evidence on the fertility and sexual needs of lymphoma survivors is scarce, limiting the capacity to direct care and support systems for this group. There is minimal evidence of the spiritual needs of this population. No included studies reported the needs of lymphoma survivors from Africa or South America. Broader literature reporting on lymphoma heavily emphasises the increased risk of infection for this group.^82^, ^83^ Yet limited literature included in this review reflects this concern.

Themes contain textured and nuanced layers, and this review has used them to capture the rich diversity of the needs of lymphoma survivors. The findings of this review extend our current understanding of lymphoma survivors who experience a myriad of unmet needs from diagnosis to survivorship.

### 4.1 Study limitations

The fundamental limitations of current literature involve the lack of qualitative, mixed methods and longitudinal approaches, with many included studies conducted at single sites, limiting generalisability. The methodological quality of included articles was high. Yet, key details relating to sociodemographic information, socioeconomic status, time since diagnosis, stage of disease and the stage of survivorship require improved reporting to improve understanding. Furthermore, the overall heterogeneity and assorted reporting of included literature have severely impaired the ability to quantify this research question through meta‐analytical approaches. In addition, there was wide‐ranging use of varying instruments to assess the needs or associated factors (i.e., quality of life outcomes) of lymphoma survivors, with only one instrument designed and validated for a lymphoma‐specific population (FACT‐Lym).

While the rapid review method is used inconsistently and with poor quality reporting in the literature,^27^ this rapid review employed several recommendations from Cochrane's interim rapid review guidance, including search strategy involvement of an information specialist and two independent reviewers for dual screening of abstracts with conflict resolution. However, it is essential to acknowledge that reviews can only be as good as the quality of the primary research on which it is based. This review cannot claim to have exhausted all relevant evidence and excluded non‐English language literature despite drawing on systematic approaches.

### 4.2 Clinical implications

Future research is required to address the sparse focus on lymphoma‐specific research and disparate reporting, which impairs a comprehensive understanding of the needs of lymphoma survivors. Moreover, the concepts of unmet needs and quality of life are closely related. This review identifies a need for further clarification on the relationship between unmet needs and quality of life to help guide future research and enhance meta‐analytical approaches in this area. This review shows that lymphoma survivors experience a myriad of unmet needs across multiple domains, reinforcing the need for lymphoma‐specific research. Therefore, the findings of this review may offer input for the development of supportive interventions for these survivors.

### 4.3 Conclusions

Despite advances in the survivorship rates of lymphoma survivors, the treatment of cancer is unfortunately not without consequence. This often contributes to various needs that are frequently long‐term. Lymphoma survivors are largely active in our global workforce yet experience a significant burden of financial and work‐related concerns. They experience disruption to psychosocial wellbeing, including fear of cancer recurrence, fragmented health services and insufficient provision of survivorship information. This review identified important and timely information which can aid the improvement of survivorship care for this growing, diverse and underserved population.

## CONFLICT OF INTEREST

The authors declare no conflicts of interest.

## ETHICAL STATEMENT

Ethical approval was not required for this review.

## Data Availability

Data sharing not applicable to this article as no datasets were generated or analysed during the current study.
